# Libraries and the future of scholarly communication

**DOI:** 10.1186/1476-4598-5-58

**Published:** 2006-11-07

**Authors:** Edwin V Sperr

**Affiliations:** 1 NELINET, Inc., 153 Cordaville Road, Southborough, MA 01772, USA

## Abstract

Changes in the structure of commercial scholarly publishing have led to spiraling subscription prices. This has resulted in a "serials crisis" that has eroded library budgets and threatened the system of scientific communication. Open access represents one possible solution, and librarians are working to help make it a reality.

Science fundamentally requires communication, and the scholarly article has been the primary medium for this communication for the past three centuries. For at least half that time, scientific communication has also relied upon libraries. No researcher alone could ever hope to own every work needed, but by pooling their resources, a *community *of scholars could acquire a great deal. However, changes in commercial publishing, as well as the very volume of information published, have combined to put this model under great strain.

The structure of scientific communication has undergone a quiet revolution over the past decade. A researcher can now do a literature search and click through to full-text articles in minutes, all without leaving her desk. It can be so effortless as to seem almost like magic, at least until she attempts to follow a citation to a journal that the library doesn't subscribe to. At that point, all magic ceases – she has the option of either waiting several days, parting with a substantial sum from her own pocket, or just throwing up her hands and looking for another article.

The fundamental problem is that the market for scholarly information is broken. Researchers use content, but are insulated from the costs, as they aren't directly responsible for the library's bills. Furthermore, the substitution of expensive titles with less expensive ones is impossible. If the article one *needs *is in journal A, then no number of articles from journal B will suffice. This all adds up to a distinct lack of pricing pressure – libraries essentially have to agree to pay whatever a publisher demands.

Commercial publishers have reacted to this market inefficiency by extracting as much money as they can. Prices for an individual title are typically in the thousands of dollars for titles in science, technology and medicine (STM), and some approach the twenty-thousand dollar mark. Publishers also often "bundle" access to weaker titles along with more popular ones, requiring libraries to pay for the whole package if they want favorable terms. As commercial scholarly publishers have consolidated through mergers, STM publishing has grown into a huge industry, one with total revenues of nearly ten billion dollars and an average profit margin of 25% [1].

Librarians have coined a term for this state of affairs: the *serials crisis*. Subscription charges have become a black hole into which the library's budget disappears. Unit costs for serials at American Research Libraries (ARL) institutions rose by some 188% percent between 1986 and 2004 [2], and journals now make up over three quarters of the average ARL library's materials budget [3].

At the same time, a smaller proportion of recorded knowledge actually makes it into libraries for scholars to use. Library purchases of books have actually *decreased *since 1986. As tight budgets have led to recurring journal cancellation projects at institutions of every size, the situation for unique journal titles is much the same [4]. This is against the backdrop of a landscape where the number of articles cited each year in PubMed increased by 150% from 1980 to 2005.

As *Molecular Cancer *is an open access journal, you are likely already aware that open access publishing represents an attractive alternative to the current system. By design, open access literature is available to all, regardless of institutional affiliation or library budget. For researchers, there is evidence to support the notion that open access directly benefits authors by making it more likely that their work will be cited by others [5]. For librarians, this model is even more compelling, as it represents a way of better meeting their patrons' needs without putting even more strain on tight resources.

It is to this end that academic libraries are at the forefront of open access activism. At many institutions, librarians seek to educate faculty members about the crisis in scholarly communications and their rights as authors. They do this either informally or increasingly through Offices of Scholarly Communications, often sited within the library. Indeed, one of the premier organizations in this effort, the Scholarly Publishing and Academic Resources Coalition (SPARC), is funded by the Association of College and Research Libraries.

There are also libraries that are supporting Open Access in more direct ways. One of the most welcome developments of the past several years has been that of the *Institutional Repository *– a place online where the scholarly output of an institution can be gathered up and shared on an Open Access basis. There are now hundreds of these systems up and running around the world, many of them managed by the institution's library. The arXiv [6], the online pre-print archive that has changed the way high-energy physicists communicate, is now housed at the Cornell University Library. Most notably, there are now even some libraries that are taking the plunge and becoming full-fledged open access publishers themselves.

Open access is beginning to take root outside of libraries as well. The NIH operates PubMed Central, a repository of open access literature, and has established a policy that requests NIH-funded scientists to contribute their manuscripts [7]. A number of commercial publishers are starting to pursue a "hybrid" open access strategy where researchers have the option of paying an additional fee to make their articles freely available. The Senate has even introduced a bill, the Federal Research Public Access Act of 2006, requiring federal agencies that fund substantial amounts of research to start *ensuring *that such work is available to taxpayers on an open access basis [8].

None of this will really have an impact however, until researchers realize the power they have and demand more open access to their own work. It is time for librarians and scholars to work together to preserve the system of scholarly communication upon which science depends.
